# Quality of diabetes care in breast, colorectal, and prostate cancer

**DOI:** 10.1007/s11764-018-0717-5

**Published:** 2018-10-06

**Authors:** Robert I. Griffiths, Emily C. McFadden, Richard J. Stevens, Jose M. Valderas, Bernadette A. Lavery, Nada F. Khan, Nancy L. Keating, Clare R. Bankhead

**Affiliations:** 1 Nuffield Department of Primary Care Health Sciences, University of Oxford, Radcliffe Primary Care Building, Radcliffe Observatory Quarter, Woodstock Road, Oxford, 0X2 6GG UK; 2 Division of General Internal Medicine, Johns Hopkins University School of Medicine, Baltimore, 21205 USA; 3 Health Services & Policy Research Group and Exeter Collaboration for Primary Care (APEx), University of Exeter, Exeter, EX4 4SB UK; 4 Oxford University Hospitals NHS Foundation Trust, Oxford, OX3 9DU UK; 5 Royal Liverpool University Hospital, Liverpool, L7 8XP UK; 6 Department of Health Care Policy, Harvard Medical School, Boston, 02115 USA; 7 Division of General Internal Medicine and Primary Care, Brigham and Women’s Hospital, Boston, 02115 USA

**Keywords:** Diabetes mellitus, Neoplasms, Primary health care, Quality of health care, Quality indicators

## Abstract

**Purpose:**

Overlooking other medical conditions during cancer treatment and follow-up could result in excess morbidity and mortality, thereby undermining gains associated with early detection and improved treatment of cancer. We compared the quality of care for diabetes patients subsequently diagnosed with breast, colorectal, or prostate cancer to matched, diabetic non-cancer controls.

**Methods:**

Longitudinal cohort study using primary care records from the Clinical Practice Research Datalink, United Kingdom. Patients with pre-existing diabetes were followed for up to 5 years after cancer diagnosis, or after an assigned index date (non-cancer controls). Quality of diabetes care was estimated based on Quality and Outcomes Framework indicators. Mixed effects logistic regression analyses were used to compare the unadjusted and adjusted odds of meeting quality measures between cancer patients and controls, overall and stratified by type of cancer.

**Results:**

3382 cancer patients and 11,135 controls contributed 44,507 person-years of follow-up. In adjusted analyses, cancer patients were less likely to meet five of 14 quality measures, including: total cholesterol ≤ 5 mmol/L (odds ratio [OR] = 0.82; 95% confidence interval [CI], 0.75–0.90); glycosylated hemoglobin ≤ 59 mmol/mol (adjusted OR = 0.77; 95% CI, 0.70–0.85); and albumin creatinine ratio testing (adjusted OR = 0.83; 95% CI, 0.75–0.91). However, cancer patients were as likely as their matched controls to meet quality measures for other diabetes services, including retinal screening, foot examination, and dietary review.

**Conclusions:**

Although in the short-term, cancer patients were less likely to achieve target thresholds for cholesterol and HbA1c, they continued to receive high-quality diabetes primary care throughout 5 years post diagnosis.

**Implications for Cancer Survivors:**

These findings are important for cancer survivors with pre-existing diabetes because they indicate that high-quality diabetes care is maintained throughout the continuum of cancer diagnosis, treatment, and follow-up.

**Electronic supplementary material:**

The online version of this article (10.1007/s11764-018-0717-5) contains supplementary material, which is available to authorized users.

## Introduction

Early detection and advances in therapy and supportive care have substantially improved the relative survival of many of the most common types of cancer [1]. Consequently, overall morbidity and mortality in cancer depend increasingly on the quality and outcomes of primary care for underlying conditions [2]. In response, Cancer Research UK (CRUK) and other cancer organizations, such as Macmillan Cancer Support, have expressed concern that overlooking other medical conditions during cancer treatment and follow-up could result in excess morbidity and mortality. This, in turn, could undermine gains associated with early detection and improved treatment of cancer [3, 4].

Although cancer could have an adverse impact on many underlying conditions, and vice-versa, the quality and outcomes of diabetes care in cancer deserve specific attention for the following reasons. First, diabetes and cancer are common, especially in older people. Second, diabetes and some types of cancer, including breast and colorectal, co-occur at rates that are higher than expected by chance alone. This implies shared risk factors and, possibly, causal mechanisms [5]. Third, some types of cancer treatments, for instance, androgen deprivation therapy for prostate cancer [6], appear to increase the risk of diabetes and related complications. Fourth, diabetes is associated with excess morbidity and mortality in cancer [7].

There have been several of studies on the quality of diabetes care in cancer patients, predominantly in the United States (US) [8–14]. However, these studies have produced inconsistent findings on the impact of cancer on the provision of medical services, laboratory testing, and control of blood pressure, cholesterol, and glycosylated hemoglobin (HbA1c). While patients in the US and United Kingdom (UK) may be clinically similar in many respects, differences in the organization, delivery, and financing of primary care services between the two countries, including historically poorer coverage of preventative services by public health insurers in the US, could undermine the generalizability of US studies to the UK.

In the UK, evidence is limited to one study based on the UK General Practice Research Database (2003–2006). In this study, long-term survivors of breast, colorectal, or prostate cancer were followed for blood pressure, cholesterol, and HbA1c monitoring and control beginning at least 5 years after cancer was diagnosed [15]. However, the study was conducted in long-term survivors, and did not assess differences in the quality of diabetes primary care during the first 5 years after cancer. This was a limitation acknowledged by the investigators, who called for research on the shorter-term consequences of cancer [15]. Also, the study did not include an assessment of other diabetes primary care services, such as retinal screening and foot examination. Furthermore, it was conducted when the Quality and Outcomes Framework (QOF)—the annual reward and incentive program detailing practice achievement results, which rewards practices for the provision of quality care and helps standardize improvement in the delivery of primary medical services—was first implemented.

The aim of this study was to compare the quality of diabetes primary care between diabetic patients subsequently diagnosed with breast, colorectal, or prostate cancer and matched, diabetic, non-cancer controls during the first 5 years after cancer diagnosis.

## Method

### Study design and data source

This was a longitudinal cohort study using primary care records from the UK Clinical Practice Research Datalink (CPRD) [16]. The CPRD contains information on demographics, symptoms, tests, diagnoses, therapies, health-related behavior, and referrals to secondary care for over 11.3 million patients from 674 general practitioner (GP) practices in the UK [17]. There are 4.4 million active (alive, currently registered) patients in the database, which is approximately 6.9% of the UK population. These patients are broadly representative of the UK general population in terms of age, sex, and ethnicity.

### Patient selection

#### Selection of cancer patients

Diabetic cancer patients were included if they met all of the following criteria: (A) diagnosed with breast, colorectal, or prostate cancer on or after January 1, 2000; (B) diagnosed with diabetes at least 2 years before their date of cancer diagnosis (index date); (C) had no other cancer diagnosis, except non-melanoma skin cancer, before their index date; (D) were age ≥ 50 years at their index date; (E) had at least 2 years of eligible CPRD data before their index date, where “eligible CPRD data” was defined as the later of their GP practice up-to-standard date or their practice registration date; (F) had an index date before the end of the eligible CPRD data, where “end of the eligible CPRD data” was defined as the earliest of death, transfer out of the GP practice, or the date of their practice’s last data upload; and (G) survived at least 1 year after cancer diagnosis. Men with breast cancer were excluded. Read codes from the Department of Health, Data and Business (QOF) Rules, Cancer and Diabetes Indicator Sets, version 25.0 [18], were used to identify diabetes and cancer in CPRD. Read codes are a standard terminology for describing the care and treatment of patients in UK primary care.

#### Selection of controls

Each diabetic cancer patient was matched to up to four diabetic, non-cancer controls on GP practice number, sex, and age (± 1 year) at cancer diagnosis. Matched controls were assigned an index date within 1 year of their cancer case, and also required to have met inclusion criteria B–G above. In addition to three separate cancer/control cohorts, cancer patients and controls were combined into a single cohort (“combined cancer cohort”).

#### Propensity score matching

For purposes of conducting sensitivity analyses, propensity-matched cohorts also were constructed from those cohorts described above. In this study, the propensity score was the conditional probability of being diagnosed with one of the three types of cancers that defined entry into the cohorts for the cancer cases. First, binary logistic regression using backwards stepwise elimination of predictors [19, 20] with a probability (*P*) value > 0.2 was used to estimate the conditional probability of “assignment” to cancer or control given the following vector of demographic and clinical covariates: demographic characteristics consisting of age at index date, sex, calendar year of index date, most recent smoking status, most recent drinking status, and Index of Multiple Deprivation (IMD) quintile, from least deprived = 1 to most deprived = 5; clinical characteristics consisting of body mass index (BMI), Charlson Comorbidity Index, history of other cardiovascular disease (atrial fibrillation, coronary heart disease, heart failure, hypertension, and stroke/transient ischemic attack), history of microvascular complications of diabetes (retinopathy, neuropathy, nephropathy, and chronic kidney disease), history of macrovascular complications of diabetes (peripheral arterial disease, acute myocardial infarction, cerebrovascular accident, and lower limb amputation), and history of anti-diabetic medications; and laboratory characteristics consisting of blood pressure (mm Hg), total cholesterol (mmol/L), and HBA1c (mmol/mol). Second, the predicted conditional probabilities of cancer from the final models were transformed into logits (ln[p/1−p]) to normalize their distributions [21]. Third, nearest neighbor matching within a caliper of 0.25σ was used to match controls 1:1 to cancer cases.

### Follow-up

Patients were followed from 2 years before, up to 5 years after, their index date. They were censored at the earliest of the following: the end of the year of follow-up preceding the one in which they died, e.g., a patient who died during the third year after their index date was censored at the end of the second year of follow-up; the end of the year of follow-up preceding the one containing their last date of eligible data; or 5 years after their index date.

### Variables

#### Primary outcome measures

Measures used to compare the quality of diabetes primary care between cancer patients and controls were based on the QOF diabetes indicator set (Box) [18].

Box: primary outcome measures

**Table:** 

• Last blood pressure reading in the year was ≤ 150/90 mmHg. (QOF DM002) • Last blood pressure reading in the year was ≤ 140/80 mmHg. (QOF DM003) • Last total cholesterol test result in the year was ≤ 5 mmol/L. (QOF DM004) • Received an albumin creatinine ratio test during the year. (QOF DM005) • Treated with an ACE-I or an ARB during the year (applies only to those diagnosed with nephropathy or microalbuminuria). (QOF DM006) • Last HbA1c test in the year was ≤ 59 mmol/mol. (QOF DM007) • Last HbA1c test in the year was ≤ 64 mmol/mol. (QOF DM008) • Last HbA1c test in the year was ≤ 75 mmol/mol. (QOF DM009) • Influenza immunization during the year. (QOF DM010) • Retinal screening during the year. (QOF DM011) • Foot examination during the year. (QOF DM012) • Dietary review during the year. (QOF DM013) • Male patient asked about erectile dysfunction during the year. (QOF DM015) • Male patient received advice/assessment of erectile dysfunction during the year. (QOF DM016)	

Since all QOF indicators are based on annual assessments, only patients alive at the end of the year were included in the “at risk” population for that year; therefore, patients who survived less than 1 year after their index date were excluded.

In order to construct clinical/test-based quality measures (blood pressure, cholesterol, and HbA1c), results of tests and blood pressure readings were obtained from 2 years before the index date until the end of the last full year (up to five) the patient remained in the study. Patients were considered to have met the quality measure in a specific year if their last reading or test result in that year was at or below the threshold specified in the corresponding QOF indicator. Patients who had no results during a specific year were considered not to have met the measure during that year. Read codes from the diabetes QOF Indicator Set [18] were used to construct quality measures based on the delivery of diabetes primary care services. Patients with no record of a specific service during a specific year were considered not to have met the quality measure in that year.

#### Covariates

Baseline demographic characteristics consisted of age at index date, sex, calendar year of index date, most recent smoking status, most recent drinking status, and IMD quintile. Baseline clinical characteristics included BMI, Charlson Comorbidity Index [22, 23], and history of microvascular and macrovascular complications of diabetes. Complications were identified using Read Codes [24] present in patient records at any time prior to the index date.

Baseline laboratory values consisted of blood pressure, total cholesterol, and HbA1c. These were identified using the most recent result within 1 year prior to the index date. Categorical variables for laboratory values were constructed using the diabetes QOF rules: blood pressure ≤ 140/80 mmHg, total cholesterol ≤ 5 mmol/L (193 mg/dL), and HbA1c ≤ 59 (7.5%), 59 to ≤ 64 (8.0%), 64 to ≤ 75 (9.0%), and > 75 mmol/mol. Baseline medications included antidiabetic agents, lipid lowering agents, and medications for blood pressure. Baseline medications were identified using British National Formulary (BNF) codes in the CPRD Therapy file [25] within 1 year prior to the index date.

Several approaches to addressing potential problems caused by missing data, including multiple imputations [26], were considered for this study. However, absence of data on important baseline variables such as smoking status, BMI, and HbA1c could have been informative, i.e., indicators of poor quality of care or lower GP contact prior to the index date. Therefore, where applicable, separate categories for missing data were constructed for baseline demographic, clinical, and treatment variables.

### Statistical methods

Multilevel logistic regression analyses were performed to estimate the unadjusted and adjusted odds of meeting each of the quality measures in cancer patients compared to controls. All covariates described in the section above (and included in the propensity score matching) were included in the adjusted analyses. In addition, test result-based quality measures were partitioned into the probability of being measured/tested, and the conditional (upon being measured/tested) probability of meeting the measure/test threshold.

#### Primary analyses

The primary analyses were conducted using up to 5 years of follow-up after the index date. Unadjusted analyses included cancer (yes/no) and time (in years) as independent variables. Adjusted analyses also included all baseline demographic and clinical characteristics (described in the previous section), as well as time, as independent variables. Furthermore, in order to examine whether the effect of cancer on outcomes changed over time, we performed an additional adjusted analysis for each outcome measure in which we also included an interaction term for cancer and time. We then compared the two models (with versus without the interaction term) for each outcome measure using the likelihood ratio test to determine whether adding the interaction term improved the fit of the model. We interpreted a statistically significant (*p* ≤ 0.05) likelihood ratio test as indicating that the effect of cancer on the outcome measure changed significantly over time.

Given the large number of multivariate analyses, adjustment for multiple comparisons was performed using the Benjamini-Hochberg [27–29] approach. *P* values from a family of comparisons (defined as the set of multivariate analyses performed on one cohort) were ordered from high to low and then compared to the corresponding Benjamini-Hochberg threshold for statistical significance.

#### Secondary analyses

Secondary analyses consisted of repeating the adjusted primary analyses using all 7 years of data including 2 years prior to the index date, and of repeating both the adjusted primary and secondary analyses using the propensity matched cohorts. Multilevel logistic regression analyses were performed in STATA 14 [30] using the xtset and xtlogit commands [31].

## Results

There were 14,517 patients in combined cohort: 3382 (23.3%) cancer patients and 11,135 (76.7%) controls (see Appendix A for characteristics). Overall, the median length of follow-up was 1274 days (3.5 years), and follow-up was statistically significantly shorter in cancer patients (median 1187 days) than controls (median 1295 days: log-rank test for equality of survivor functions *p* < 0.0001).

**Fig. 1:**
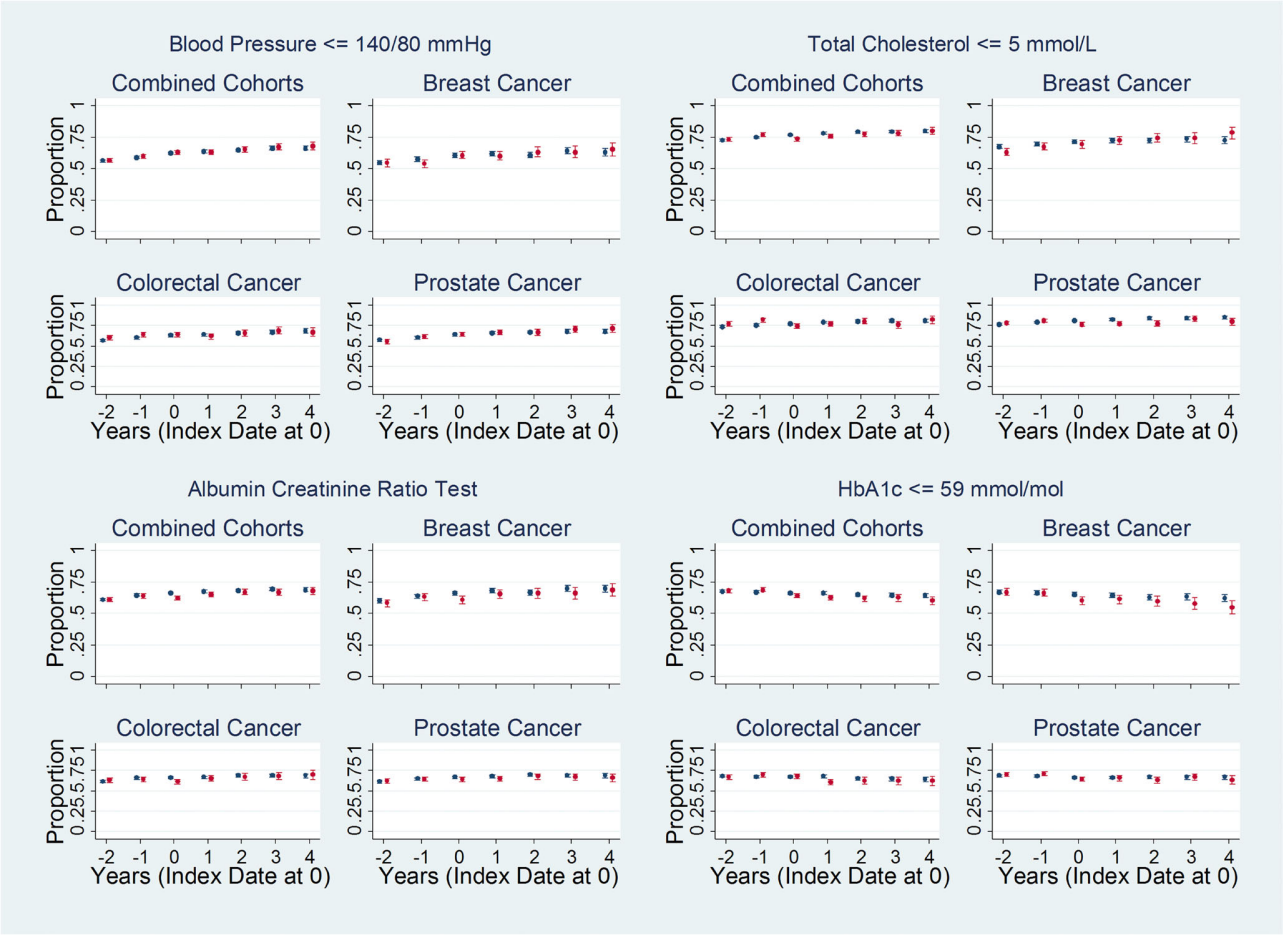
Unadjusted proportions of patients meeting quality measures based on testing. Unadjusted proportions (dots) and 95% confidence intervals (bars) of cancer patients (red) and controls (blue) meeting select quality measures based on testing (Fig. 1) and services (Fig. 2), from 2 years before, up to 5 years after, the date of cancer diagnosis or matched date in the control group (index date). Plots for other quality measures not shown here (blood pressure ≤ 150/90 mmHg, diagnosis of nephropathy or micro-albuminuria who were treated with an angiotensin converting enzyme inhibitor or angiotensin receptor blocker, HbA1c ≤ 64 mmol/mol, HbA1c ≤ 75 mmol/mol, asked about erectile dysfunction, received advice about erectile dysfunction) are provided in Appendix B

**Fig. 2:**
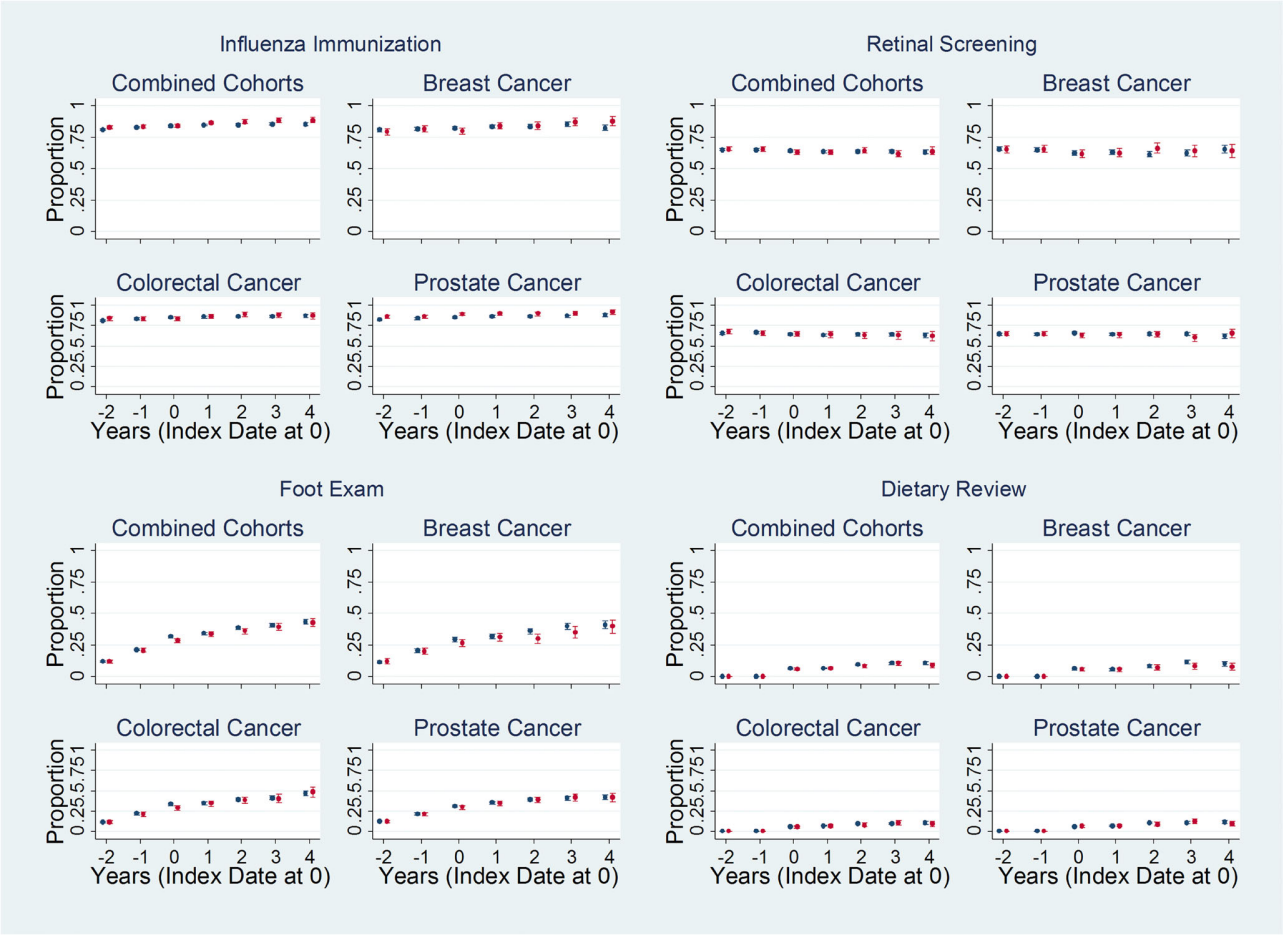
Unadjusted proportions of patients meeting quality measures based on services. Unadjusted proportions (dots) and 95% confidence intervals (bars) of cancer patients (red) and controls (blue) meeting select quality measures based on testing (Fig. 1) and services (Fig. 2), from 2 years before, up to 5 years after, the date of cancer diagnosis or matched date in the control group (index date). Plots for other quality measures not shown here (blood pressure ≤ 150/90 mmHg, diagnosis of nephropathy or micro-albuminuria who were treated with an angiotensin converting enzyme inhibitor or angiotensin receptor blocker, HbA1c ≤ 64 mmol/mol, HbA1c ≤ 75 mmol/mol, asked about erectile dysfunction, received advice about erectile dysfunction) are provided in Appendix B

Unadjusted plots of the annual proportions of patients meeting the quality measures suggest that, compared to non-cancer controls, (A) slightly lower proportions of prostate cancer patients achieved cholesterol control (total cholesterol ≤5 mmol/L), (B) lower proportions of breast and colorectal cancer patients achieved HbA1c control (HbA1c ≤ 59 mmol/mol), and (C) lower proportions of breast and colorectal cancer patients received an albumin creatinine ratio test—at least during the first year after cancer diagnosis (Fig. 1). In contrast, higher proportions of prostate cancer patients received influenza immunization (Fig. 2). There were no discernable differences between the proportions of cancer patients and controls meeting other quality measures for retinal screening, foot examination, dietary review (Fig. 2), ACE-I or ARB use for nephropathy/micro-albuminuria, or those for erectile dysfunction (presented in Appendix B, since they applied only to select subsets of the study population).

### Primary analyses

The results of the primary multilevel logistic regression analyses (Table 1) show that in the combined cohort, after adjustment for baseline characteristics and for multiple comparisons, cancer patients were statistically significantly (*p* ≤ 0.05) less likely than non-cancer controls to meet five of 14 quality measures examined, including: total cholesterol ≤5 mmol/L (adjusted OR = 0.82; 95% CI, 0.75–0.90); HbA1c ≤ 59 mmol/mol (adjusted OR = 0.77; 95% CI, 0.70–0.85); and albumin creatinine ratio testing (adjusted OR = 0.83; 95% CI, 0.75–0.91). Cancer patients were statistically significantly more likely than non-cancer controls to receive influenza immunization (adjusted OR = 1.31; 95% CI, 1.07–1.59), and to get advice about erectile dysfunction. Cancer patients were as likely as their matched controls to meet quality measures for other diabetes services, including retinal screening, foot examination, and dietary review.

**Table 1:** Unadjusted and adjusted odds ratios (cancer compared with control) of meeting quality measures

Quality measure	Cohort							
	Combined		Breast cancer		Colorectal cancer		Prostate cancer	
	Unadjusted^a^	Adjusted^b^	Unadjusted	Adjusted	Unadjusted	Adjusted	Unadjusted	Adjusted
Blood pressure ≤ 150/90 mmHg								
Odds ratio^c^	1.02	1.05	0.96	1.00	0.96	0.96	1.14	1.16*
95% CI	(0.93–1.13)	(0.96–1.16)	(0.81–1.14)	(0.85–1.18)	(0.81–1.14)	(0.81–1.13)	(0.97–1.13)	(1.00–1.35)
Blood pressure ≤ 140/80 mmHg								
Odds ratio	1.02	1.04	1.00	1.06	1.00	0.99	1.04	1.06
95% CI	(0.94–1.10)	(0.97–1.12)	(0.87–1.15)	(0.93–1.20)	(0.88–1.14)	(0.87–1.11)	(0.92–1.18)	(0.95–1.19)
Total cholesterol ≤ 5 mmol/L								
Odds ratio	0.81***^†^	0.82***^†^	0.95	1.03	0.85	0.80**^†^	0.66***^†^	0.66***^†^
95% CI	(0.73–0.89)	(0.75–0.90)	(0.80–1.15)	(0.88–1.21)	(0.72–1.01)	(0.68–0.93)	(0.57–0.77)	(0.57–0.76)
Albumin creatinine ratio test								
Odds ratio	0.81***^†^	0.83***^†^	0.78**^†^	0.80**	0.81*	0.81*	0.84*	0.86
95% CI	(0.73–0.89)	(0.75–0.91)	(0.65–0.92)	(0.68–0.95	0.67–0.97)	(0.68–0.97)	(0.72–0.99)	(0.73–1.01)
ACE-I or ARB^d^								
Odds ratio	0.64	0.48	0.85	0.57^↑^	0.81	0.76^↑^	0.44	0.33^↑^
95% CI	(0.30–1.35)	(0.21–1.10)	(0.13–5.75)	(0.05–5.98)	(0.26–2.60)	(0.23–2.56)	(0.14–1.43)	(0.09–1.14)
HbA1c ≤ 59 mmol/mol								
Odds ratio	0.80***^†^	0.77***^†^	0.74**	0.72***^†^	0.79*	0.80*^↑^	0.86	0.79**^†^
95% CI	(0.72–0.89)	(0.70–0.85)	(0.60–0.90)	(0.61–0.85)	(0.65–0.97)	(0.68–0.95)	(0.72–1.02)	(0.68–0.92)
HbA1c ≤ 64 mmol/mol								
Odds ratio	0.78***^†^	0.75***^†^	0.76**^†^	0.74***^†^	0.76**	0.77**^†^	0.80*	0.73**^†^
95% CI	(0.70–0.87)	(0.68–0.82)	(0.62–0.93)	(0.63–0.88)	(0.63–0.93)	(0.65–0.91)	(0.67–0.95)	(0.63–0.85)
HbA1c ≤ 75 mmol/mol								
Odds ratio	0.81***^†^	0.80***^†^	0.86	0.86	0.74**	0.76**	0.83*	0.79**^†^
95% CI	(0.73–0.91)	(0.73–0.89)	(0.70–1.05)	(0.72–1.03)	(0.60–0.90)	(0.63–0.91)	(0.70–1.00)	(0.67–0.93)
Influenza immunization								
Odds ratio	1.26*	1.31**^†↑^	0.93	0.95^↑^	0.98	0.96^↑^	2.04***^†^	2.18***^†^
95% CI	(1.03–1.53)	(1.07–1.59)	(0.66–1.31)	(0.68–1.34)	(0.69–1.39)	(0.67–1.36)	(1.46–2.86)	(1.56–3.06)
Retinal screening								
Odds ratio	0.96	0.99	1.01	1.03	1.00	1.03	0.89	0.91
95% CI	(0.88–1.05)	(0.91–1.08)	(0.87–1.18)	(0.88–1.19)	(0.85–1.18)	(0.88–1.20)	(0.77–1.03)	(0.79–1.05)
Foot exam								
Odds ratio	0.79**^†^	0.94	0.75*	0.88	0.76*	0.92	0.93	1.03
95% CI	(0.68–0.92)	(0.85–1.04)	(0.57–0.98)	(0.74–1.04)	(0.58–0.99)	(0.78–1.09)	(0.71–1.21)	(0.87–1.21)
Dietary review								
Odds ratio	0.85	1.01	0.77	0.89	0.87	1.02	0.98	1.08
95% CI	(0.70–1.03)	(0.87–1.16)	(0.53–1.13)	(0.66–1.21)	(0.66–1.14)	(0.80–1.28)	(0.69–1.39)	(0.83–1.41)
Asked about erectile dysfunction								
Odds ratio	0.94	1.06	NA	NA	0.86	1.00	1.02	1.08
95% CI	(0.77–1.16)	(0.89–1.26)	NA	NA	(0.64–1.15)	(0.75–1.32)	(0.78–1.34)	(0.86–1.35)
Advice about erectile dysfunction								
Odds ratio	1.55*	1.60**	NA	NA	1.17	1.26	1.68**	1.71**^†^
95% CI	(1.09–2.19)	(1.18–2.18)	NA	NA	(0.65–2.11)	(0.71–2.26)	(1.14–2.48)	(1.21–2.41)

*ACE-I* angiotensin-converting enzyme inhibitor, *ARB* angiotensin receptor blocker, *HbA1c* glycosylated hemoglobin, *NA* not applicable

*p < 0.05, **p < 0.01, ***p < 0.001

^†^Remained statistically significant after Benjamini-Hochberg adjustment for multiple comparisons

^↑^Goodness of fit of the model was significantly improved by adding an interaction term for time versus cancer, and interaction term indicates effect of cancer over time was to increase the adjusted odds of the outcome measure in the cancer group

^a^The multilevel logistic regression models for the unadjusted odds ratios also included time (years) as a factor variable and a random effect for the patient

^b^The regression models for the adjusted odds ratios also included age, sex (combined cohorts, colorectal cancer), year of index date, smoking status, drinking status, body mass index, Charlson Comorbidity Index, Index of Multiple Deprivation score, baseline blood pressure, baseline total cholesterol, baseline HbA1c, history of one or more microvascular complications of diabetes, history of one or more macrovascular complications of diabetes, and history of diabetes medications

^c^Odds ratios: cancer compared to control

^d^In patients diagnosed with nephropathy or microalbuminuria

Of note, adjusted odds ratios for the three HbA1c indicators, as well as those for the two blood pressure indicators, were similar (Table 1). This indicates that it is unlikely cancer patients had slightly higher thresholds than the controls, which were missed simply because the binary measures were not sufficiently sensitive to detect small differences between the two groups. Also, there were few instances in which adding an interaction for cancer and time significantly improved the goodness of fit of the adjusted model (Table 1), indicating that the impact of cancer on the outcome measures did not change over time.

In some instances, the impact of cancer on the quality of diabetes primary care differed across the three individual cancer cohorts. For example, in the breast cancer cohort, the adjusted OR for total cholesterol ≤ 5 mmol/L was 1.03 (95% CI, 0.88–1.21), but in prostate cancer it was 0.66 (95% CI, 0.57–0.76). In the breast cancer cohort, the adjusted OR for influenza immunization was 0.95 (95% CI, 0.68–1.34), but in prostate cancer it was 2.18 (95% CI, 1.56–3.06). In other instances, findings were consistent across the cohorts.

Findings from the analyses in which the test-based quality measures were partitioned into (A) being tested (yes/no) in that year, and (B) meeting the test result threshold for that quality measure (yes/no) conditional upon having been tested are reported in Appendix C. The results indicate that overall differences between cancer patients and controls were due to a combination of differences in testing and meeting the test thresholds; however, this varied by type of cancer.

### Secondary analyses

Comparison of demographic and clinical characteristics, as well as laboratory values and medications, showed substantial improvement in the balance of individual characteristics between cancer patients and controls after propensity score matching. After matching, there were no statistically significant differences in the distributions of any categorical variables. However, small differences between cancer patients and controls also remained in mean diastolic blood pressure (0.52 mmHg higher in cancer) and in total cholesterol (0.03 mmol/L higher in cancer). Finally, a comparison of mean logit scores (propensity scores transformed by ln [P/1-P] to normalize the distributions) between cancer patients and controls confirmed no statistically significant difference after matching (*p* = 0.51).

Findings from the multivariate analyses based on the propensity matched cohorts, and those that included 2 years of data prior to cancer diagnosis or the matched index date for controls, were consistent with those from the primary analyses. (Appendix D).

## Discussion

Leading cancer organizations in the UK have expressed concern that overlooking other conditions during cancer treatment and follow-up could undermine improvements in survival among cancer patients, due to advances in early detection, treatment, and supportive care [3, 4]. Our results show that cancer patients who survived at least 1 year after diagnosis were up to 34% less likely to meet quality measures for testing and control of total cholesterol and HbA1c during the first 5 years after diagnosis. These findings were consistent across the three cancer cohorts (except cholesterol in breast cancer). Also, they were robust to varying the length of the observation period, and to changing the composition of the cohorts. There was no evidence that cancer was associated with lower adjusted odds of blood pressure control, or with receiving any of the other services in the Diabetes Mellitus Indicator Set, except perhaps for foot examination.

Prostate cancer was associated with statistically significantly higher adjusted odds of receiving influenza immunization. Prostate cancer also was associated with statistically significantly higher adjusted odds of receiving advice for erectile dysfunction. One possible explanation for these findings is that follow-up of prostate cancer, with the aims of checking how cancer has responded to treatment, and helping patients manage any side effects of treatment, increases the frequency of appointments at the patient’s GP surgery, which in turn provides more opportunities for providing routine primary care, such as influenza immunization, to these patients. In particular, regular prostate specific antigen testing may be performed in the primary care setting, with subsequent appointments to review the results. Also, since GPs will be aware that erectile dysfunction is a side-effect of treatment for prostate cancer, and patients are encouraged to discuss symptoms or treatment side-effects during their follow-up visits, prostate cancer patients are more likely to receive advice about erectile dysfunction.

Compared to Khan et al. [15], who investigated the impact of cancer on testing and disease control in long-term UK cancer survivors beginning 5 years after cancer diagnosis, cancer had less adverse impact on the odds of monitoring and testing in our study. Of note, we found no impact on blood pressure monitoring within 5 years after cancer diagnosis. Whereas Khan and colleagues found breast and prostate cancer patients were less likely to receive monitoring beginning 5 years after cancer. Also, in our study, cancer was associated with poorer control of cholesterol and HbA1c. Specifically, the adjusted odds of meeting the HbA1c test threshold of ≤ 59 mmol/mol, which equates to < 7.5% used by Khan and colleagues, were statistically significantly lower in all three cancer cohorts versus only colorectal cancer patients in the earlier study. Further, the adjusted odds of meeting the total cholesterol test threshold were lower in colorectal and prostate cancer versus only prostate cancer in the aforementioned study.

Our study has several strengths, which are in large part attributable to the high quality of the database we used. Since the study was based on CPRD we were able to control for additional clinical factors typically not available in US health insurance claim databases, which may have otherwise confounded associations between cancer and the quality of diabetes primary care outcome measures. These included baseline BMI, smoking status, blood pressure, cholesterol, and HbA1c. As most of the differences we observed between cancer patients and controls occurred in the test result-based quality measures, we believe that controlling for baseline test values (at the same cut points as the quality measures) in the multivariate analyses has removed an important source of potential confounding. Second, the study design and analytical approach allowed us to investigate within-person changes in the adjusted odds of meeting the quality measures. Although we cannot rule out the occurrence of other contemporaneous (with the diagnosis of cancer) events, the fact that the results of the seven-year analyses (including 2 years before the index date) were virtually identical to those of the 5-year analyses gives us greater confidence that cancer caused the differences in quality measures we observed between cases and controls. Third, since all of our quality measures were based on QOF performance indicators, there are considerable incentives for primary care practices to report the delivery of all services and results pertaining to these indicators.

Our study also has several limitations. First, at the time it was conducted we were unable to link the CPRD data to information from the National Cancer Intelligence Network. This would have given us details on cancer stage and initial treatment. Although primary care data have a high sensitivity and specificity for identifying cancer [32], registry data would have allowed us to exclude cancer patients diagnosed with metastatic disease. We considered using Read codes in the primary care data files or International Classification of Diseases, 10th Revision (ICD-10) codes in the Hospital Episode Statistics (HES) inpatient data to stage patients. However, we are not aware of any studies in the UK validating the use of ICD-10 codes for this purpose, and since only two thirds of patients in our study were linked to HES doing so would have limited our sample sizes. Instead, we excluded patients who died within the first year after their index date. This approach could have resulted in including cancer patients with relatively poor prognosis, for whom cancer care would be prioritized over diabetes care; inclusion of such patients could create a bias towards finding less receipt of recommended diabetes care among cancer patients. Second, we considered several approaches to missing data. We elected to construct additional categories for missing values of predictor variables because we believed in this instance “missingness” might be informative, and possibly negatively correlated with receiving diabetes primary care services. Third, we could not account for diabetes services performed in secondary care settings during cancer treatment that were not recorded in the primary care data. Fourth, the measures of biological control, i.e., blood pressure, cholesterol, and HbA1c, that we included in the study were binary variables based on threshold values in the QOF diabetes indicator set [18]. These thresholds have been established as indicators of high-quality diabetes care, and they are part of the UK “pay for performance” system for GP practices. However, an alternative (and perhaps more sensitive) approach might have been to use the actual laboratory values. Finally, our findings may not be generalizable either to long-term survivors of breast, colorectal, and prostate cancer in an era of full implementation of QOF, or to other types of cancers and primary care services for other comorbid conditions.

## Conclusions

Overall, cancer appears to have had little adverse impact on the delivery of high-quality diabetes primary care services during the first 5 years after cancer diagnosis. However, cancer patients were less likely to achieve target thresholds for cholesterol and HbA1c. Potential reasons, including the effects of cancer treatment and changes in patient health behaviors in response to cancer diagnosis, should be further investigated. Options to improve achievement of target thresholds could include the development of specific indicators and incentives to promote greater coordination of care between oncologists and general practitioners, especially to identify and address instances where cancer treatment may cause blood sugar and/or cholesterol levels to rise, and the expansion of diabetes programs that are tailored specifically to cancer patients.

## Electronic supplementary material

Appendix A (DOCX 29 kb)

Appendix B (DOCX 49 kb)

Appendix C (DOCX 29 kb)

Appendix D (DOCX 1223 kb)

## References

[1] Cancer Research UK. Cancer survival for common cancers. Available from: http://health-pr.com/health-professional/cancer-statistics/survival/common-cancers-compared#undefined (accessed 11 January 2017).

[2] Ning Y, Shen Q, Herrick K, et al. Cause of death in cancer survivors. Cancer Res. 2012;72(8 Suppl):Abstract nr LB-339.

[3] Cancer Research UK. Longer cancer survival means nearly half of cancer patients die from other diseases. Available from: http://www.cancerresearchukorg/about-us/cancer-news/news-report/2012-04-03-longer-cancer-survival-means-nearly-half-of-cancer-patients-die-from-other-diseases?view=rss (accessed 11 January 2017).

[4] Macmillan Cancer Support. Throwing light on the consequences of cancer and its treatment July 2013. Available from: http://wwwncsiorguk/wp-content/uploads/MAC14312_CoT_Throwing-light_report_FINALpdf (accessed 1 February 2016).

[5] Giovannuci E, Harlan DM, Archer MC (2010). Diabetes and cancer: a consensus report. Diabetes Care.

[6] Keating NL, O’Malley AJ, Smith MR (2006). Diabetes and cardiovascular disease during androgen deprivation therapy for prostate cancer. J Clin Oncol.

[7] Barone BB, Yeh H-C, Snyder CF, Peairs KS, Stein KB, Derr RL, Wolff AC, Brancati FL (2008). Long-term all-cause mortality in cancer patients with preexisting diabetes mellitus: a systematic review and meta-analysis. JAMA.

[8] Snyder CF, Frick KD, Herbert RJ, Blackford AL, Neville BA, Wolff AC, Carducci MA, Earle CC (2013). Quality of care for comorbid conditions during the transition to survivorship: differences between cancer survivors and noncancer controls. J Clin Oncol.

[9] Irizarry L, Li QE, Duncan I, Thurston AL, Fitzner KA, Edwards BJ, McKoy-Bent JM, Tulas KM, McKoy JM (2013). Effects of cancer comorbidity on disease management: making the case for diabetes education (a report from the SOAR program). Popul Health Manag.

[10] Bayliss EA, Blatchford PJ, Newcomer SR, Steiner JF, Fairclough DL (2011). The effect of incident cancer depression and pulmonary disease exacerbations on type 2 diabetes control. J Gen Intern Med.

[11] Chiao EY, Nambi PV, Naik AD (2010). The impact of diabetes process and outcome quality measures on overall survival in patients with co-morbid colorectal cancer. J Cancer Surviv.

[12] Hanchate AD, Clough-Gorr KM, Ash AS, Thwin SS, Silliman RA (2010). Longitudinal patterns in survival comorbidity healthcare utilization and quality of care among older women following breast cancer diagnosis. J Gen Intern Med.

[13] Keating NL, Zaslavsky AM, Herrinton LJ, Selby JV, Wolf RE, Ayanian JZ (2007). Quality of diabetes care among cancer survivors with diabetes. Med Care.

[14] Earle CC, Neville BA (2004). Under use of necessary care among cancer survivors. Cancer.

[15] Khan NF, Mant D, Rose PW (2010). Quality of care for chronic diseases in a British cohort of long-term cancer survivors. Ann Fam Med.

[16] Clinical Practice Research Datalink. Available from: https://wwwcprdcom/introasp (accessed 11 January 2017).

[17] Herrett E, Gallagher AM, Bhaskaran K, Forbes H, Mathur R, van Staa T, Smeeth L (2015). Data resource profile: clinical practice research datalink (CPRD). Int J Epidemiol.

[18] Primary Care Commissioning. Available from: http://wwwpcc-cicorguk/article/qof-business-rules-v250 (accessed 11 January 2017).

[19] Rosenbaum PR (2002). Observational studies.

[20] Rosenbaum PR, Rubin DB (1984). Reducing bias in observational studies using subclassification on the propensity score. J Am Stat Assoc.

[21] Guo S, Fraser MW (2010). Propensity score analysis: statistical methods and applications advanced quantitative techniques in the social sciences 12.

[22] Charlson ME, Pompei P, Ales KL, MacKenzie CR (1987). A new method of classifying prognostic comorbidity in longitudinal studies: development and validation. J Chronic Dis.

[23] Khan NF, Perera R, Harper S, Rose PW (2010). Adaptation and validation of the Charlson Index for Read/OXMIS coded databases. BMC Fam Pract.

[24] Kontopantelis E, Springate DA, Reeves D, Ashcroft DM, Rutter M, Buchan I, Doran T (2015). Glucose blood pressure and cholesterol levels and their relationships to clinical outcomes in type 2 diabetes: a retrospective cohort study. Diabetologia.

[25] Dave S, Petersen I (2009). Creating medical and drug code lists to identify cases in primary care databases. Pharmacoepidemiol Drug Saf.

[26] Marston L, Carpenter JR, Walters KR, Morris RW, Nazareth I, Petersen I (2010). Issues in multiple imputation of missing data for large general practice clinical databases. Pharmacoepidemiol Drug Saf.

[27] Thissen D, Steinberg L, Kuang D (2002). Quick and easy implementation of the Benjamini-Hochberg procedure for controlling the false positive rate in multiple comparisons. J Educ Behav Stat.

[28] Samuel CA, Landrum MB, McNeil BJ, Bozeman SR, Williams CD, Keating NL (2014). Racial disparities in cancer care in the veterans affairs health care system and the role of site of care. Am J Public Health.

[29] Williams VSL, Jones LV, Tukey JW (1999). Controlling error in multiple comparisons with examples from state-to-state differences in educational achievement. J Educ Behav Stat.

[30] StataCorp (2015). Stata statistical software: release 14.

[31] Rabe-Hesketh S, Skrondal A (2012). Multilevel and longitudinal modeling using Stata; volume II: categorcial responses counts and survival.

[32] Boggon R, van Staa TP, Chapman M, Gallagher AM, Hammad TA, Richards MA (2013). Cancer recording and mortality in the general practice research database and linked cancer registries. Pharmacoepidemiol Drug Saf.

