# Supplementary material for: Quality of diabetes care in breast, colorectal, and prostate cancer
Source: J Cancer Surviv. 2018 Oct 6;12(6):803–12. doi: 10.1007/s11764-018-0717-5 (PMC6244927; doi:10.1007/s11764-018-0717-5)
Supplement: Supplementary file 1 — (DOCX 29 kb) [file 11764_2018_717_MOESM1_ESM.docx]

Patient Characteristics

|  | Combined Cohort | | | | | |  |
| --- | --- | --- | --- | --- | --- | --- | --- |
|  | Cancer (n=3,382) | | Control (n=11,135) | | All (n=14,517) | |  |
|  | n | % | n | % | n | % | P-Value |
| Age |  |  |  |  |  |  |  |
| 50-<60 | 248 | 7.3 | 800 | 7.2 | 1,048 | 7.2 | 0.740 |
| 60-<70 | 946 | 28.0 | 3,098 | 27.8 | 4,044 | 27.9 |  |
| 70-<80 | 1,498 | 44.3 | 5,041 | 45.3 | 6,539 | 45.0 |  |
| ≥80 | 690 | 20.4 | 2,196 | 19.7 | 2,886 | 19.9 |  |
| Sex |  |  |  |  |  |  |  |
| Male | 1,974 | 58.4 | 6,551 | 58.8 | 8,525 | 58.7 | 0.630 |
| Female | 1,408 | 41.6 | 4,584 | 41.2 | 5,992 | 41.3 |  |
| Year of Diagnosis |  |  |  |  |  |  |  |
| 2000-2004 | 546 | 16.1 | 1,649 | 14.8 | 2,195 | 15.1 | 0.087 |
| 2005-2009 | 1,500 | 44.4 | 4,902 | 44.0 | 6,402 | 44.1 |  |
| ≥2010 | 1,336 | 39.5 | 4,584 | 41.2 | 5,920 | 40.8 |  |
| Smoking Status |  |  |  |  |  |  |  |
| Non Smoker | 991 | 29.3 | 3,364 | 30.2 | 4,355 | 30 | <0.001 |
| Ex Smoker | 1,716 | 50.7 | 5,633 | 50.6 | 7,349 | 50.6 |  |
| Current Smoker | 324 | 9.6 | 1,240 | 11.1 | 1,564 | 10.8 |  |
| Not Reported | 351 | 10.4 | 898 | 8.1 | 1,249 | 8.6 |  |
| Body Mass Index |  |  |  |  |  |  |  |
| <25 | 579 | 17.1 | 1,932 | 17.4 | 2,511 | 17.3 | <0.001 |
| 25-<30 | 1,272 | 37.6 | 4,183 | 37.6 | 5,455 | 37.6 |  |
| ≥30 | 1,344 | 39.7 | 4,656 | 41.8 | 6,000 | 41.3 |  |
| Not Reported | 187 | 5.5 | 364 | 3.3 | 551 | 3.8 |  |
| Charlson Comorbidity Index |  |  |  |  |  |  |  |
| 1-2 | 1,878 | 55.5 | 5,898 | 53.0 | 7,776 | 53.6 | 0.024 |
| 3-4 | 1,044 | 30.9 | 3,578 | 32.1 | 4,622 | 31.8 |  |
| >4 | 460 | 13.6 | 1,659 | 14.9 | 2,119 | 14.6 |  |
| Type of Cancer (or Control) |  |  |  |  |  |  |  |
| Breast | 1,036 | 30.6 | 3,194 | 28.7 | 4,230 | 29.1 | <0.001 |
| Colorectal | 1,069 | 31.6 | 4,047 | 36.3 | 5,116 | 35.2 |  |
| Prostate | 1,277 | 37.8 | 3,894 | 35.0 | 5,171 | 35.6 |  |
| Type of Diabetes |  |  |  |  |  |  |  |
| Type I | 141 | 4.2 | 554 | 5.0 | 695 | 4.8 | 0.054 |
| Type II | 3,241 | 95.8 | 10,581 | 95.0 | 13,822 | 95.2 |  |
|  |  |  |  |  |  |  |  |
| Any Microvascular Complication |  |  |  |  |  |  |  |
| No | 2,412 | 71.3 | 7,750 | 69.6 | 10,162 | 70.0 | 0.056 |
| Yes | 970 | 28.7 | 3,385 | 30.4 | 4,355 | 30.0 |  |
| Any Macrovascular Complication |  |  |  |  |  |  |  |
| No | 2,681 | 79.3 | 8,684 | 78.0 | 11,365 | 78.3 | 0.113 |
| Yes | 701 | 20.7 | 2,451 | 22.0 | 3,152 | 21.7 |  |
| Any Antidiabetic Agent |  |  |  |  |  |  |  |
| No | 769 | 22.7 | 2,363 | 21.2 | 3,132 | 21.6 | 0.060 |
| Yes | 2,613 | 77.3 | 8,772 | 78.8 | 11,385 | 78.4 |  |
| Blood Pressure ≤ 140/80 mm Hg |  |  |  |  |  |  |  |
| Yes | 2,026 | 59.9 | 6,770 | 60.8 | 8,796 | 60.6 | <0.001 |
| No | 1,250 | 37.0 | 4,252 | 38.2 | 5,502 | 37.9 |  |
| Not Reported | 106 | 3.1 | 113 | 1.0 | 219 | 1.5 |  |
|  |  |  |  |  |  |  |  |
| Systolic mean(SD) | 3,276 | 136(15) | 11,022 | 136(16) | 14,298 | 136(16) | 0.205 |
|  |  |  |  |  |  |  |  |
| Diastolic mean(SD) | 3,276 | 74(9) | 11,022 | 74(9) | 14,298 | 74(9) | 0.090 |
| Total Cholesterol ≤ 5mmol/L (193mg/dL) |  |  |  |  |  |  |  |
| Yes | 2,706 | 80.0 | 8,971 | 80.6 | 11,677 | 80.4 | <0.001 |
| No | 475 | 14.0 | 1,826 | 16.4 | 2,301 | 15.9 |  |
| Not Reported | 201 | 5.9 | 338 | 3.0 | 539 | 3.7 |  |
|  |  |  |  |  |  |  |  |
| mean(SD) in mmol/L | 3,181 | 4.2(0.94) | 10,797 | 4.3(1.57) | 13,978 | 4.2(1.45) | <0.001 |
| mean in mg/dL |  | 162.4 |  | 166.3 |  | 162.4 |  |
| HbA1c(mmol/mol) |  |  |  |  |  |  |  |
| ≤59 (7.5%) | 2,179 | 64.4 | 6,995 | 62.8 | 9,174 | 63.2 | 0.362 |
| 59-≤64 (8.0%) | 310 | 9.2 | 1,054 | 9.5 | 1,364 | 9.4 |  |
| 65-≤75 (9.0%) | 322 | 9.5 | 1,074 | 9.6 | 1,396 | 9.6 |  |
| >75 | 185 | 5.5 | 702 | 6.3 | 887 | 6.1 |  |
| Not Reported | 386 | 11.4 | 1,310 | 11.8 | 1,696 | 11.7 |  |
|  |  |  |  |  |  |  |  |
| mean(SD) | 2,996 | 54.0(12.5) | 9,825 | 54.5(13.1) | 12,821 | 54.3(13.0) | 0.060 |
| mean (%) |  | 7.1 |  | 7.1 |  | 7.1 |  |

SD Standard Deviation; HbA1c Glycosylated haemoglobin
