# Supplementary material for: Quality of diabetes care in breast, colorectal, and prostate cancer
Source: J Cancer Surviv. 2018 Oct 6;12(6):803–12. doi: 10.1007/s11764-018-0717-5 (PMC6244927; doi:10.1007/s11764-018-0717-5)
Supplement: Supplementary file 2 — (DOCX 49 kb) [file 11764_2018_717_MOESM2_ESM.docx]

**Legend:** Adjusted odds ratios and 95% confidence intervals (cancer compared to controls) of undergoing blood pressure measurement, cholesterol testing, and HbA1c testing, from the multivariate mixed effects logistic regression analyses of the full cohorts during five years after cancer diagnosis or matched date in controls. Upper bound of the 95% confidence interval < 1.0 indicates cancer patients had lower odds of being measured/tested than controls. Lower bound of the 95% confidence interval > 1.0 indicates cancer patients had higher odds of being measured/tested than controls.

**Legend:** Adjusted odds ratios and 95% confidence intervals (cancer compared to controls) of having a measurement/test result at or below the threshold for meeting the corresponding quality measure (shown on x axis), given that the patient was measured/tested. Results are from the multivariate mixed effects logistic regression analyses of the full cohorts during five years after cancer diagnosis or matched date in controls. Upper bound of the 95% confidence interval < 1.0 indicates cancer patients had lower odds of meeting the threshold than controls. Lower bound of the 95% confidence interval > 1.0 indicates cancer patients had higher odds of meeting the threshold than controls.

*blood pressure ≤ 140/80 mm Hg, **total cholesterol ≤ 5 mmol/L,

***HbA1c ≤ 59 mmol/mol
