# Supplementary material for: Quality of diabetes care in breast, colorectal, and prostate cancer
Source: J Cancer Surviv. 2018 Oct 6;12(6):803–12. doi: 10.1007/s11764-018-0717-5 (PMC6244927; doi:10.1007/s11764-018-0717-5)
Supplement: Supplementary file 3 — (DOCX 29 kb) [file 11764_2018_717_MOESM3_ESM.docx]

Adjusted Odds (Cancer Compared to Control) of Being Measured or Tested

Adjusted Odds (Cancer Compared to Control) of Meeting Thresholds.
