# Supplementary material for: Quality of diabetes care in breast, colorectal, and prostate cancer
Source: J Cancer Surviv. 2018 Oct 6;12(6):803–12. doi: 10.1007/s11764-018-0717-5 (PMC6244927; doi:10.1007/s11764-018-0717-5)
Supplement: Supplementary file 4 — (DOCX 1223 kb) [file 11764_2018_717_MOESM4_ESM.docx]

Secondary Analysis: Adjusted Odds (Cancer Compared to Control) of Meeting Quality Measures

Full cohorts, seven years of observation (including two years before index date)

Propensity matched cohorts, five years of observation (after index date)

Propensity matched cohorts, seven years of observation (including two years before index date)
